# A survey of genetic fetal-haemoglobin modifiers in Nigerian patients with sickle cell anaemia

**DOI:** 10.1371/journal.pone.0197927

**Published:** 2018-06-07

**Authors:** Titilope A. Adeyemo, Oyesola O. Ojewunmi, Idat A. Oyetunji, Helen Rooks, David C. Rees, Adebola O. Akinsulie, Alani S. Akanmu, Swee Lay Thein, Stephan Menzel

**Affiliations:** 1 Department of Haematology and Blood Transfusion, College of Medicine, University of Lagos, Idi-araba, Lagos, Nigeria; 2 Sickle Cell Foundation Nigeria, Idi-araba, Lagos, Nigeria; 3 School of Cancer and Pharmaceutical Sciences, King’s College London, London, United Kingdom; 4 King's College Hospital, Paediatric Haematology, London, United Kingdom; 5 Department of Paediatrics, College of Medicine, University of Lagos, Idi-araba, Lagos, Nigeria; 6 Sickle Cell Branch, National Heart Lung and Blood Institute, National Institute of Health, Bethesda, MD, United States of America; Universidade Nova de Lisboa Instituto de Higiene e Medicina Tropical, PORTUGAL

## Abstract

Genetic variants at three quantitative trait loci (QTL) for fetal haemoglobin (HbF), *BCL11A*, *HBS1L-MYB* and the β-globin gene cluster, have attracted interest as potential targets of therapeutic strategies for HbF reactivation in sickle cell anaemia (SCA). We carried out the first systematic evaluation of critical single nucleotide polymorphisms at these disease modifier loci in Nigerian patients with SCA. Common variants for *BCL11A* and *HBS1L-MYB* were strongly associated with HbF levels. At both loci, secondary association signals were detected, illustrating the mapping resolution attainable in this population. For *BCL11A*, the two independent sites of association were represented by *rs1427407* (primary site, p = 7.0 x 10^−10^) and *rs6545816* (secondary site, conditioned on *rs1427407*: p = 0.02) and for *HBS1L-MYB* by *rs9402686* (*HMIP-2B*, p = 1.23 x 10^−4^) and *rs66650371* (*HMIP-2A*, p = 0.002). Haplotype analysis revealed similarities in the genetic architecture of *BCL11A* and *HBS1L-MYB* in Nigerian patients. Variants at both loci also alleviated anaemia. The variant allele for the γ globin gene promoter polymorphism *XmnI-HBG2* was too infrequent in our patients to be evaluated in this relatively small study. Studying the large and diverse SCA patient populations in African countries such as Nigeria will be key for a clearer understanding of how these loci work and for the discovery of new disease modifier genes.

## Introduction

Sickle cell anaemia (SCA), though a monogenic disorder, is highly clinically-diverse. Part of this diversity derives from the variable genetic background of patients, and several of the factors underlying this have been identified [1]. Significant genetic disease modifiers are a co-inheritance of α-thalassemia (the α-3.7 globin gene deletion) [2] and the presence of fetal-haemoglobin (HbF) inducing genotypes at the three major quantitative-trait loci (QTL) for HbF persistence [3–8]: *XmnI-HBG2*, *BCL11A* and *HMIP*. Co-inheritance of α-thalassemia is associated with reduced haemolytic events in sickle cell patients, due to decreased intracellular concentrations of the defective haemoglobin (HbS) and thus a decreased likelihood of HbS polymerisation. Elevated levels of HbF are associated with increased life expectancy, reduced incidence of painful crisis and fewer leg ulcers [9–11]. Accordingly, the three HbF QTL have been shown to affect measures of disease pathology and severity [12–18].

Findings from genetic studies can provide guidance for new therapeutic approaches [19, 20]. Studying the large number of SCA patients residing in African countries such as Nigeria, the country with the largest SCA patient population world-wide, promises not only the discovery of new SCA modifier genes and therapeutic targets. It will also provide a better understanding of the known loci, their biological function and clinical significance. Here we are presenting the first systematic evaluation of the known HbF modifier loci in a Nigerian SCA patient population. Our data, obtained from 260 patients, give an initial estimate of the prevalence of critical HbF modifier variants at the three QTL and of their genetic architecture in Nigerian patients, providing a starting point for subsequent large-scale population-genetic studies.

### Patients and methods

260 SCA patients (138 male, 122 female) were recruited from paediatric and adult sickle cell clinics of Lagos University Teaching Hospital between March and October 2015, with a median age of 13 years (range 5–46). Patients were excluded if they were younger than 5 years, of Hb SC genotype, admitted to hospital, treated with hydroxyurea or had blood transfusion three months prior to the study. The diagnosis was confirmed in all patients using high performance liquid chromatography (Bio-Rad D-10; Bio-Rad Laboratories, Hercules, CA, USA). This method cannot distinguish between Hb SS and Hb S/β^0^ genotypes. Since the two are phenotypically very similar, the fact that a few patients must have the latter genotype can be disregarded.

The study protocol was approved by the Health Research Ethics Committee of Lagos University Teaching Hospital (ADM/DCST/HREC/1686). Written informed consent was obtained from the patients and parents/guardians prior to study enrolment. Children who were ≥ seven years old gave assent to participate in the study. A study proforma was completed to obtain the socio-demographic and clinical data for all the study participants. A total of 7 ml blood sample was taken from each patient for haematological, biochemical and genotyping assays. The full blood count (data summarised in S1 Table) was determined using an automated haematology analyser (Mindray, BC-2800).

DNA was extracted using the phenol-chloroform method. Seven single nucleotide polymorphisms tagging trait-relevant genetic variability at the three HbF modifier loci (*BCL11A*, *HBS1L-MYB*, and *XmnI-HBG2*) were genotyped. *rs6545816* and *rs1427407* of *BCL11A* and *rs9376090*, *rs66650371*, *rs9402686* and *rs6920211* of *HMIP-2* were assayed using TaqMan chemistry, as previously described [21]. The assay for *XmnI-HBG2* (*rs7482144*) was performed after PCR-amplification specifically of *HBG2* promoter sequence, omitting the homologous *HBG1* area [22]. Genotypes for all markers were in Hardy-Weinberg equilibrium, except *rs7482144*, which is in linkage with the sickle mutation.

Genetic association analysis was performed by multiple regression, with age and sex as covariates (SPSS v. 15). Blood counts data were log-transformed to normalize them. Haplotypes were constructed from the patient genotypes with Haploview 4.2 [23].

1000 Genomes project, Phase III, data [24] were accessed at http://phase3browser.1000genomes.org. Phase-aligned variant call format (vcf) files were downloaded, covering chromosome 2 from position (hg19) 60,710,000 to 60,730,000.

## Results

**Fig 1:**
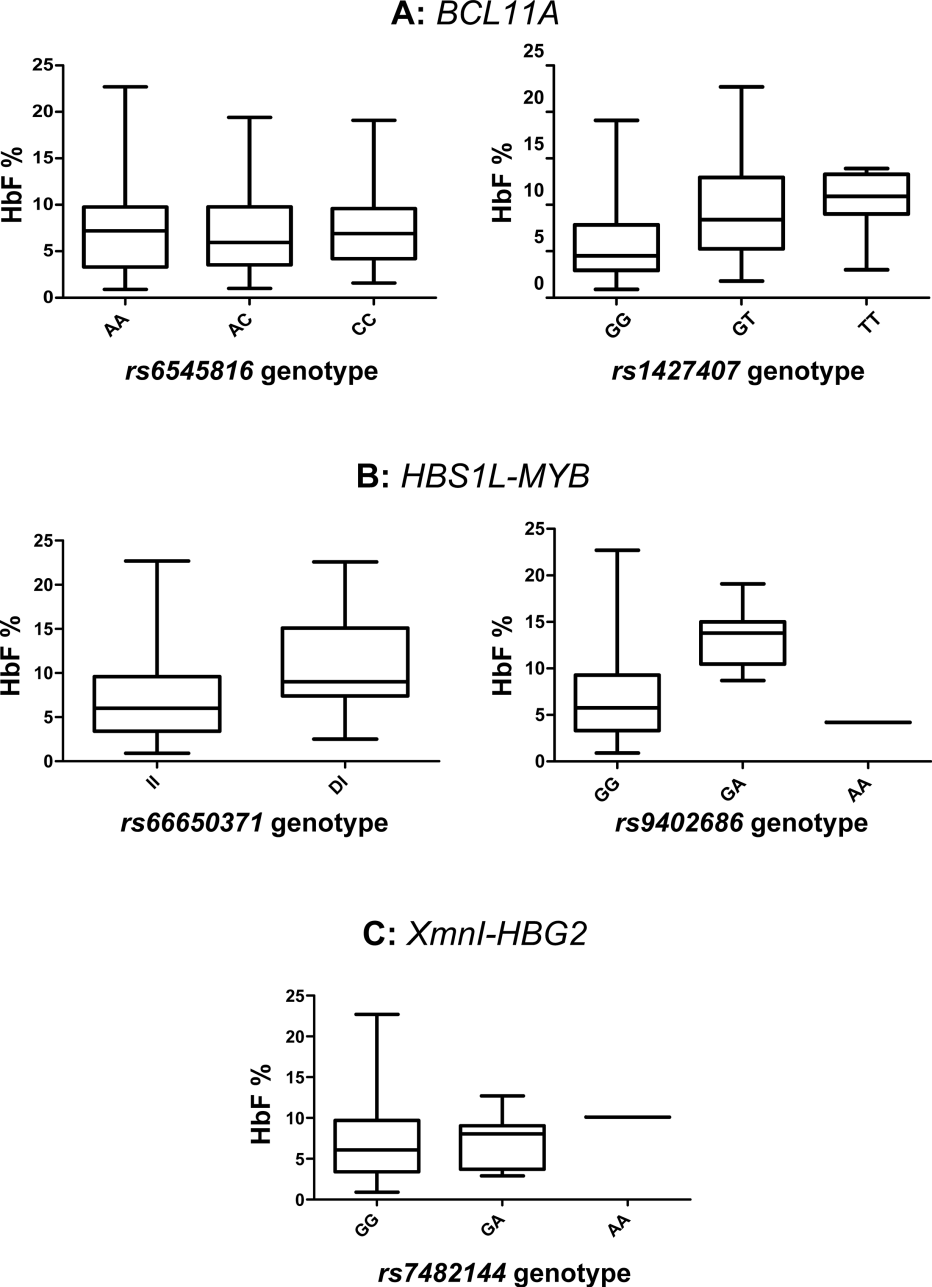
Genotypic values for HbF levels at the three main QTL. Boxes show the inter-quartile range; the line denotes the median. Whiskers indicate the full range of values observed. P-values are shown in Table 2.

**Fig 2:**
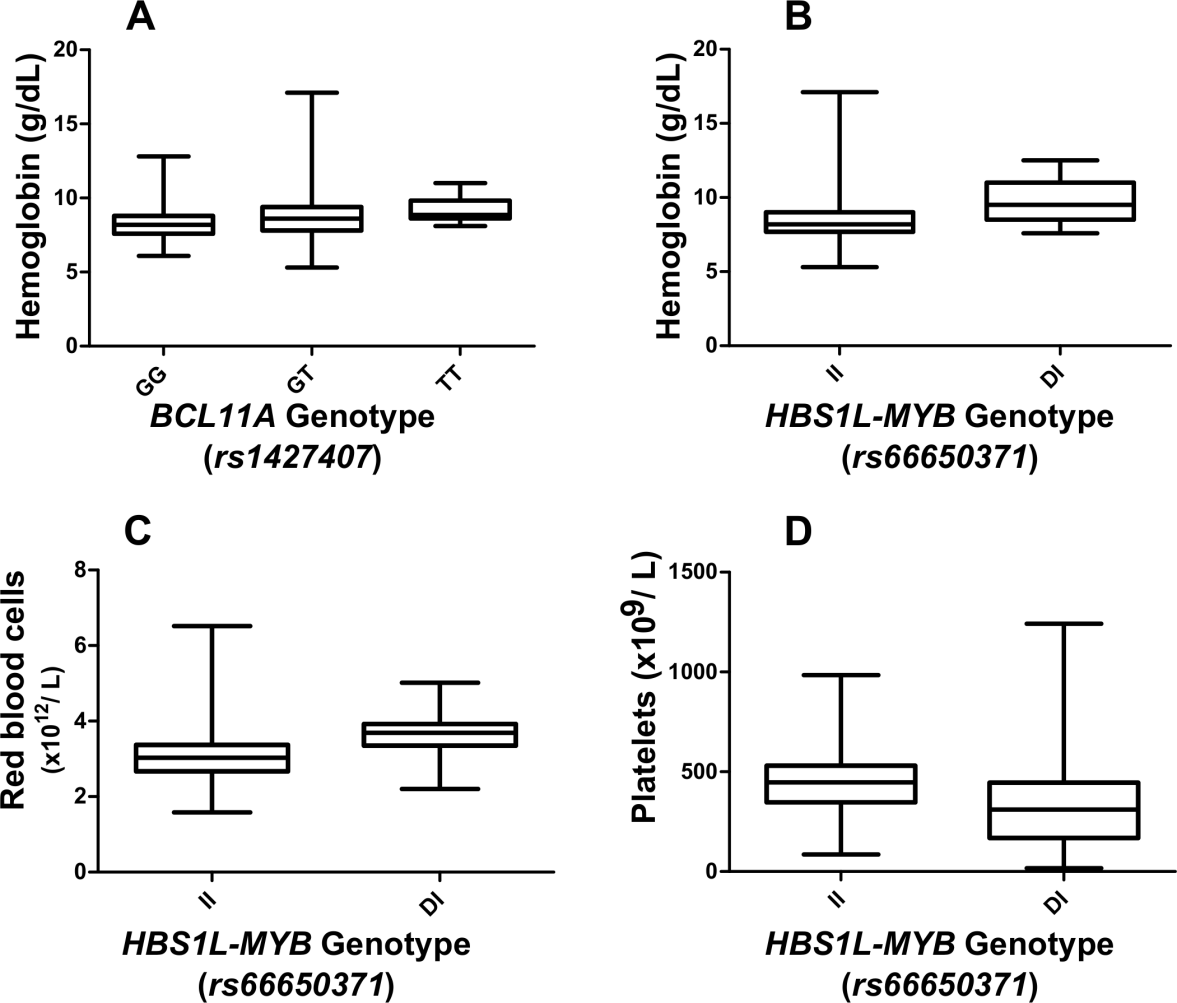
Variants with significant (p < 0.005) impact on haematological variables in our patients. Boxes show the inter-quartile range; the line denotes the median. Whiskers indicate the full range of values observed. Individual p-values are shown in Table 2.

Seven key genetic variants, *rs6545816* and *rs1427407* (*BCL11A)*, *rs9376090*, *rs66650371*, *rs9402686* and *rs6920211* (*HBS1L-MYB)* and *rs7482144* (*XmnI-HBG2)* were genotyped in 260 Nigerian SCA patients (summary data shown in Table 1) and their effects on HbF levels and other haematological parameters were investigated. The median HbF% of the cohort was 6.2 (inter-quartile range 3.42–9.70). The results of our genetic-association analysis are shown in Table 2 and genotypic values for HbF and other haematological parameters are plotted in Figs 1 and 2.

**Table 1:** Presence and frequency of HbF-boosting genetic variants in Nigerian patients.

Locus	Variants	Position on chromosome	Allele change	Genotypes detected	HbF-boosting allele (frequency)
**Chromosome 2**					
BCL11A	rs6545816	60,568,365	A > C	AA, n = 97	C (35%)
				AC, n = 116	
				CC, n = 27	
	rs1427407	60,571,547	G > T	GG, n = 133	T (23%)
				GT, n = 89	
				TT, n = 8	
**Chromosome 6**					
HMIP-2	rs9376090	135,452,920	T > C	TT, n = 260	C (0%)
	rs66650371	135,460,326- 135,460,328	In > Del	II, n = 245 DI, n = 15	D (3%)
	rs9402686	135,469,509	G > A	GG, n = 244	A (3%)
				GA, n = 14	
				AA, n = 1	
	rs6920211	135,473,011	T > C	CC, n = 37	C (36%)
				TC, n = 113	
				TT, n = 109	
**Chromosome11**					
*Xmn1-HBG2*	rs7482144	5,232,745	G > A	GG, n = 247	A (2%)*
				GA, n = 8	
				AA, n = 1	

* While this variant has been found associated with HbF in other populations, we have not detected this effect in our patients.

**Table 2:** Effect of fetal haemoglobin itself and of the genetic HbF modifier variants studied on haematological outcome variables.

Variables							
	Hb F (ln HbF%)	rs6545816	rs1427407	rs66650371	rs9402686	rs6920211 (P-value)	rs7482144 (P-value)
In HbF%	——	˗ 0.013 (0.851)	**0.474 (7.04x 10**^**−10**^**)**	**0.577 (0.002)**	**0.631 (1.23 x 10**^**−4**^**)**	**0.147 (0.017)**	0.200 (0.326)
In Hb	**0.05 (7.75 x 10**^**−5**^**)**	0.01 (0.387)	**0.05 (0.004)**	**0.14 (0.001)**	0.02 (0.548)	0.002 (0.905)	0.032 (0.715)
In WBC	**-0.093 (0.001)**	0.06 (0.057)	-0.05 (0.168)	0.01 (0.906)	-0.12 (0.115)	0.02 (0.458)	0.010 (0.820)
In RBC	-0.0001 (0.997)	0.144 (0.508)	0.03 (0.284)	**0.18 (3.15 x 10**^**−4**^**)**	-0.02 (0.626)	-0.01 (0.713)	0.01 (0.931)
In PLT	**-0.08 (0.047)**	**0.131 (0.003)**	**-0.122 (0.020)**	**-0.434 (2.08 x 10**^**−4**^**)**	-0.15 (0.144)	-0.05 (0.229)	-0.09 (0.484)
MCV	**4.87 (6.70 x 10**^**−7**^**)**	-0.47 (0.584)	1.89 (0.064)	-2.86 (0.212)	**4.502 (0.031)**	0.79 (0.306)	0.44 (0.862)
In MCH	0.02 (0.191)	0.01 (0.746)	0.01 (0.615)	-0.06 (0.253)	0.04 (0.431)	0.03 (0.071)	0.01 (0.933)
MCHC	-0.19 (0.133)	0.12 (0.412)	0.15 (0.374)	-0.50 (0.187)	-0.30 (0.389)	-0.06 (0.634)	0.20 (0.628)
ln Abs. Lymphocytes	**-0.097 (0.004)**	-0.06 (0.121)	-0.07 (0.119)	-0.15 (0.139)	-0.05 (0.557)	0.02 (0.560)	-0.08 (0.468)
ln Abs. Neutrophils	**-0.12 (0.001)**	0.06 (0.147)	-0.06 (0.271)	-0.096 (0.387)	-0.13 (0.184)	-0.03 (0.386)	0.12 (0.324)
Reticulocytes	-0.35 (0.316)	-0.03 (0.934)	**-0.979 (0.033)**	-0.34 (0.740)	0.002 (0.998)	0.13 (0.694)	-1.07 (0.456)

Linear multiple regression included sex and age as covariates. Allelic effects are presented as regression coefficient (β, p-values in brackets). Bold font indicates statistical significance.

**Fig 3:**
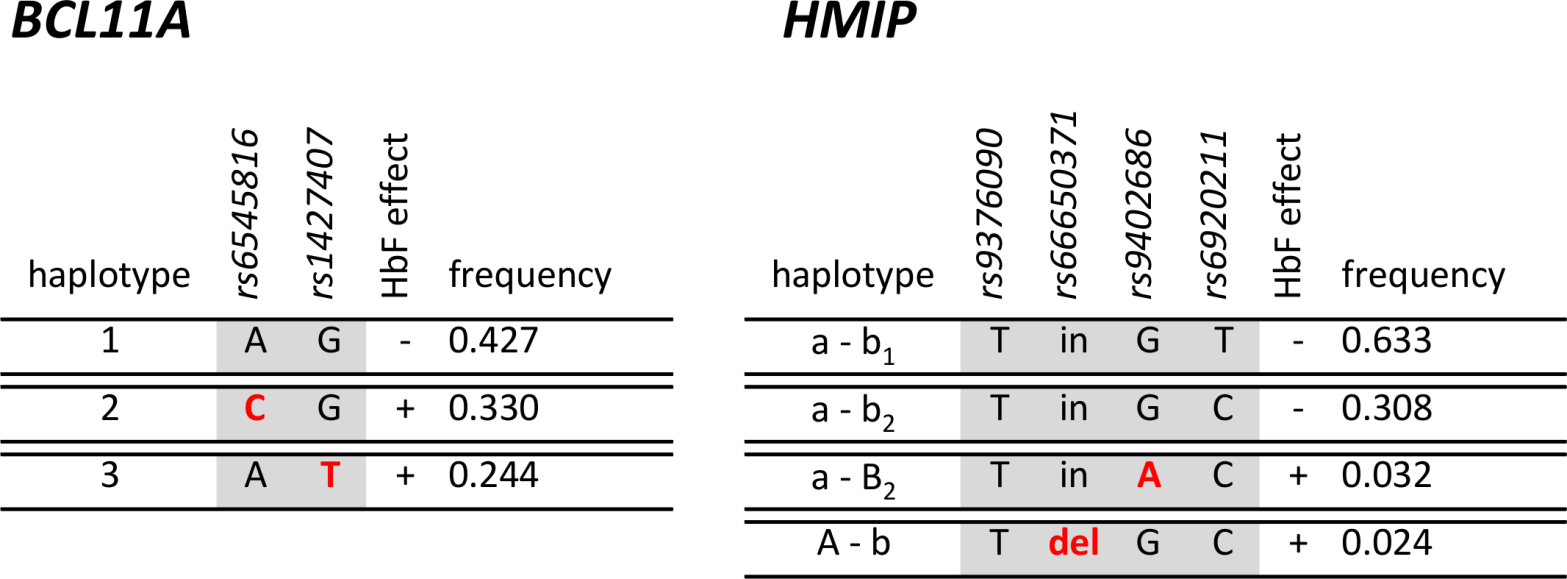
Haplotypes of genetic variants detected at the BCL11A and HMIP loci. Red letters denote HbF-increasing alleles. HMIP haplotypes were named to match the locus architecture described previously [16, 26]. A situation with two HbF-raising variants in cis, i.e. occupying the same haplotype, was not observed.

*BCL11A*: The primary variant tagging this locus, *rs1427407*, was strongly associated with HbF levels (β = 0.47, p = 7 x 10^−10^) and also showed a marked influence on anaemia: median [Hb] was 8.2 g/dL for GG genotype patients, 8.6 g/dL for the GT genotype, and 8.9 g/dL for the TT genotype [*p* = 0.011] (Fig 2). The influence of this marker is enhanced by the high prevalence of the HbF-increasing allele ‘T’ (23%), an allele frequency typical for African populations [24] and African patients with SCA [25]. As previously observed in African American [13] and Tanzanian patients [25], a second association signal at *BCL11A* (*rs6545816*) was detected when adjusting for the effect of the primary signal at *rs1427407* (Table 3). Aligning alleles at the two variants into haplotypes (Fig 3) reveals the genetic architecture underlying this finding: the HbF-boosting allele (‘C’) for *rs6545816* occurred solely on haplotypes/chromosomes carrying the *low*-HbF allele for the primary marker, *rs1427407*. Investigating human population data from the 1000 Genomes Project Phase III [24], we found that the HbF-boosting alleles for both markers were exclusively in such a *repulsion* phase alignment in African and Asian populations and that *coupling* phase (both high-HbF alleles united in *cis*) was exceedingly rare (found in 3 out of 5,008 individuals) across all human populations studied.

**Table 3:** Joint analysis of the BCL11A variants *rs6545816* and *rs1427407*.

Variables		
	*rs6545816* (conditioned on *rs1427407*)	*rs1427407* (conditioned on *rs6545816*)
In Hb F%	**0.16(0.022)**	**0.55 (6.0 x 10**^**−11**^**)**
In Hb	0.03 (0.093)	**0.07 (0.001)**
In WBC	0.05 (0.177)	-0.03 (0.478)
In RBC	0.02 (0.468)	0.03 (0.203)
In PLT	**0.12 (0.015)**	-0.07 (0.25)
MCV	0.14 (0.889)	1.96 (0.081)
In MCH	0.01 (0.529)	0.02 (0.474)
MCHC	0.27 (0.095)	0.28 (0.134)
ln Abs. Lymphocytes	-0.05 (0.333)	0.05 (0.262)
ln Abs. Neutrophils	0.04 (0.390)	-0.04 (0.514)
Reticulocytes	-0.17 (0.697)	**-1.05 (0.035)**

*HBS1L-MYB*: Both known main HbF sub-loci in this region, *HMIP-2A* (tagged by *rs66650371*) [16, 27] and *HMIP-2B* (tagged by *rs9402686*) [13, 16, 26] were significantly (p = 0.002 and p = 1.23 x 10^−4^, respectively) associated with HbF%, with similar allelic effects (β ~ 0.6). The 3-bp deleted allele of *rs66650371* was also associated with increased haemoglobin levels. It was the only variant studied that had a significant effect on the red blood cell count. This marker was also strongly associated with lower platelet counts (Table 2, Fig 2). HbF-increasing alleles at *HBS1L-MYB* had low frequencies (3%), as is characteristic for African populations. Similar to the *BCL11A* locus, HbF-increasing alleles at the two sub-loci occurred within different haplotypes (Fig 2), which is typical for individuals of African descent [13, 16, 26]. This stands in contrast to the situation in European populations, where HbF-increasing alleles usually appear to be combined into a single haplotype (*HMIP-2AB*) [16]. Accordingly, for an ancestry informative marker tagging this haplotype, *rs9376090*, we did not detect the ‘G’ allele, indicating the absence of *HMIP-2AB* haplotypes and suggesting a lack of European, Asian, or North African admixture in our patient cohort [16, 24, 26]. Of all variants studied, *rs9402686* had the largest allelic effect on HbF levels [β = 0.631, *p* = 1.23 x 10^−4^], resulting in median HbF values of 5.75% for the GG genotype and 13.8% for the GA genotype (the single person with AA genotype had an HbF of 4.2%) (Fig 1).

*XmnI-HBG2 (**rs7482144)*: As it is typical for African populations, the *rs7482144* ‘A’ allele that is associated with boosting HbF is infrequent (2%) among our patients. While a strong effect for this variant was seen in Tanzanian patients [15], we detected no association with HbF or general haematological parameters, most likely due to the small number of our patients that carry the ‘A’ allele, resulting in a lack of statistical power.

HbF levels correlated positively with total haemoglobin [β = 0.05, *p* = 7.75 x 10^−5^] and MCV [β = 4.87, *p* = 6.70 x 10^−7^] but negatively with WBC [β = -0.093, *p* = 0.001] and platelet counts [β = -0.08, *p* = 0.047].

## Discussion

In our survey of an initial group of 260 Nigerian patients with sickle cell anaemia, we have detected the effect of two known QTL for the expression of fetal haemoglobin, *BCL11A* and *HMIP*, but not for the third, *Xmn1-HBG2*. HbF-inducing variants at the former two loci showed beneficial effects on sickle cell pathology, as seen through an improvement of anaemia and other haematological variables.

Our genetic findings have identified starting points for identifying further functional DNA segments and biological mechanisms involved in the regulation of HbF expression. At *HBS1L-MYB*, the small deletion *rs66650371*, residing within the *HMIP-2A* sub-locus, is already well characterized and is likely of direct functional significance [27, 28] for critical regulatory elements within the core enhancer for *MYB*, which encodes an important erythroid transcription factor [29]. The strong association signal we obtained at the second sub-locus [13, 16], *HMIP-2B* (*rs9402686*), is providing an opening for the discovery of a novel functional site regulating HbF levels. At *BCL11A*, the primary associated variant *rs1427407* [5, 6, 14, 17] has been shown to disrupt a critical element (‘-58’) at the erythroid enhancer for this gene [30], which encodes a transcriptional repressor of γ globin gene (i.e., HbF) expression. The presence of the previously described secondary association signal (*rs6545816*) [13, 25] in Nigerian patients will help to uncover other HbF-raising alleles or regulatory elements affecting HbF levels through the *BCL11A* mechanism. Our observation that, at both loci, HbF-raising variants do not exist in *cis* in our patients (Fig 3) and in African populations in general could mean either that they are allelic, i.e. mutually-exclusive on the physical level of DNA sequence, or that natural selection has disfavoured a situation where two HbF-raising variants affect the same copy of either gene.

In our patients, we detected no effect of the ^G^γ chain promoter polymorphism *Xmn1-HBG2* on HbF levels or other haematological parameters, in contrast to what has been reported for patients from US, Tanzania, the UK and Brazil [14, 15, 18], but in agreement with findings from another West African country, Cameroon [18]. Nigerian patients have been found previously to lack [31, 32] HbF-increasing β globin gene locus haplotypes containing the ‘*Xmn1-HBG2 A’* allele (‘Arab-Indian’ and ‘Senegal’ haplotypes). Accordingly, the small number of our patients (n = 5) carrying the HbF-boosting ‘A’ allele (called *Xmn1*’+’ in older papers) has not allowed us to evaluate its effect. Other active components of the ‘Senegal’ and ‘Arab-Indian’ haplotypes have been proposed [13] and might have a more important role in Nigerian patients.

Beneficial effects of elevated HbF seen in our study (reduced anaemia, leucocytosis, and thrombocytosis) are partially explained by the genetic variants investigated here. However as observed before [14, 33] [34], the relationship between common genetic variation, HbF levels and disease phenotype is not straightforward and to unravel their mutual dependence will require a systematic dissection in large collaborative studies.

## Conclusion

The present study demonstrated the presence and beneficial effects of two quantitative-trait loci for fetal haemoglobin expression, *BCL11A* and *HMIP*, and the likely absence of a third, *Xmn1-HBG2*, in Nigerian patients with SCA. Our results make a case for the development of further, extended studies in Nigeria, as presently planned by us and others. Ideally, these will include genome-wide association testing to discover novel disease modifier loci. Up to now, most research on sickle cell disease has taken place in the US and Europe while the great majority of patients live in Africa. Genetic and epidemiological studies can help to address this imbalance.

## Supporting information

S1 Table Haematological/Biochemical characteristics of patients. (DOC)
