# Supplementary material for: A survey of genetic fetal-haemoglobin modifiers in Nigerian patients with sickle cell anaemia
Source: PLoS One. 2018 Jun 7;13(6):e0197927. doi: 10.1371/journal.pone.0197927 (PMC5991720; doi:10.1371/journal.pone.0197927)
Supplement: S1 Table — (DOC) [file pone.0197927.s001.doc]

**Supporting Information**

**S1 Table.** **Haematological/biochemical characteristics of patients**

| Parameter, n=260 | Median(IQR)/ mean ± SD |
| --- | --- |
| PCV | 25.10 (22.73 - 27.70) |
| Hb | 8.3 (7.70 - 9.10) |
| WBC | 11.90 (9.6 - 14.60) |
| RBC | 3.05 (2.67 - 3.44) |
| PLT | 441.0 (341.0 - 530.0) |
| MCV | 82.57±8.85 |
| MCH | 27.90 (25.40 - 29.80) |
| MCHC | 33.31±1.42 |
| Absolute LYMPH | 4.6 (3.52 - 5.97) |
| Absolute Neutrophils | 5.95 (4.72 - 7.67) |
| %Hb F | 6.20 (3.42 – 9.70) |
| %Retics, n=231 | 8.79±3.71 |
| LDH, n=217 | 879.1 (701.2 – 979.6) |
| AST, n=217 | 33.10 (21.05 – 46.95) |
| Creatinine, n=217 | 57.49 (46.23 – 82.12) |
| ALT, n=215 | 6.10 (3.0 – 10.30) |
